# Causes and Metabolic Consequences of Gynecomastia in Adult Patients

**DOI:** 10.1155/2019/6718761

**Published:** 2019-10-03

**Authors:** Ralitsa Robeva, Atanaska Elenkova, Sabina Zacharieva

**Affiliations:** USHATE “Acad. Iv. Penchev”, Department of Endocrinology, Faculty of Medicine, Medical University-Sofia, Sofia, Bulgaria

## Abstract

**Background:**

Gynecomastia (GM) is a benign enlargement of male breast due to glandular tissue proliferation. GM is a symptom of systemic or local hormonal disturbances, which could be associated with functional changes or pathological conditions. However, the long-lasting steroid imbalance in men with GM might exert negative influence on their metabolic health.

**Methods:**

A total of 110 adult men with symptomatic GM were included in the present retrospective cross-sectional study. Anthropometric, metabolic, and hormonal data of the patients were collected.

**Results:**

In almost 64% of GM patients, the underlying pathological condition was identified. Moreover, the development of GM was among the primary symptoms leading to the proper diagnosis in more than 40% of hypogonadal patients. The prevalence of metabolic syndrome (MS) was 53%; the highest prevalence of MS was found in patients with medication-induced GM and in the hypogonadal patients, whereas the lowest prevalence was observed in men with idiopathic postpubertal GM despite the similar degree of obesity. The lower testosterone levels were associated with more unfavorable lipid profile in the GM patients.

**Conclusion:**

The development of GM in adults might be an important symptom of an underlying gonadal disease. Moreover, it could be associated with an increased risk of metabolic disturbances. Our results support the need of detailed laboratory and hormonal investigations in patients with GM including targeted screening for metabolic disturbances. Further longitudinal studies are needed to evaluate the long-term consequences of sex hormones imbalance on cardiovascular morbidity and mortality in adults with GM.

## 1. Introduction

Gynecomastia (GM) is a benign unilateral or bilateral enlargement of male breast, which results from glandular tissue proliferation [1, 2]. The histology and growth potential of mammary gland is similar in both sexes during early development [3]. After the onset of puberty, synergetic effects of growth hormone, insulin-like growth factor 1, and estrogens ensure complete breast development in females, whereas increased androgens suppress breast development in males [4, 5]. GM might develop at any age in case of a hormonal imbalance [3]. Its prevalence peaks during mid puberty and decreases thereafter, in parallel with the completion of sexual maturation and normalization of estrogen to androgen ratio [6, 7]. The temporary breast enlargement in adolescent boys is usually benign, but a thorough physical examination is required to exclude additional signs and symptoms suggesting of an underlying disorder [8].

In the adulthood, the newly developed GM is often a symptom of a pathological condition [3]. Increased estrogens, decreased androgens, changes in sex hormone-binding globulin levels, and steroid receptor defects might be involved in the pathogenesis of GM associated with different diseases, such as hormone-secreting tumors, hypogonadism, hyperprolactinemia, hyperthyroidism, chronic liver impairment, and androgen-resistance syndromes [9]. Approximately one-quarter of the adult men develop idiopathic GM in the absence of apparent hormonal disturbances, probably, because of local hormonal changes or tissue receptor alterations [3, 10].

Because GM is a symptom of systemic or local hormonal disturbances, the detailed endocrine investigation of the patients seems reasonable despite the existing controversy on this topic [11]. Moreover, the long-lasting steroid imbalance in men with GM might exert negative influence on their metabolic profile, which could be related to increased cardiovascular risk. Men with overt atherosclerotic disease and lower testosterone to estradiol ratio have shown increased risk for major cardiovascular events in comparison to male individuals with normal sex hormone ratio [12]. However, the possible associations between the presence of GM and the development of metabolic disturbances have not been thoroughly investigated. Therefore, the present retrospective study aims to investigate the causes for GM in adult patients attending endocrinology clinic and to estimate the prevalence of metabolic syndrome in GM of pathological or idiopathic origin.

## 2. Methods

### 2.1. Participants and Study Protocol

Medical records of all adult Caucasian men referred to Endocrinology Department, Medical University‐Sofia, because of symptomatic GM or diagnosed with GM during clinical evaluation in the period 2008–2018 were initially selected (*n* = 144). A total of 22 men were excluded because of pseudogynecomastia, whereas another 12 men were excluded because of GM spontaneous regression. Thus, 110 patients were finally included in the presented retrospective cross-sectional study. Data concerning GM type (unilateral or bilateral) and duration, anthropometric (height, weight, waist circumference/WC/) and biochemical characteristics (blood count, fasting glucose/Glu/, creatinine, liver enzymes, high-density lipoprotein cholesterol/HDL/, triglycerides/TG/, and total cholesterol), blood pressure, use of medication, anabolic steroids and nutritional supplements, concomitant diseases, and hormonal values (testosterone/T/, luteinizing hormone/LH/, follicle-stimulating hormone/FSH/, estradiol/E2/, thyroid-stimulating hormone/TSH/, and prolactin/Prol/) were collected retrospectively.

A presence of firm subareolar glandular tissue determined by palpation was considered GM, as in other studies [6, 11]. In case of uncertainty or suspicion of mammary gland carcinoma, an ultrasound and/or radiological investigation was accomplished additionally (*n* = 64). Alpha-fetoprotein and human chorionic gonadotropin were also investigated, if tumor origin of GM was suspected. Testicular volume was determined by palpation; however, in case of hypogonadism, hyperestrogenemia, or suspected testicular tumor, testicular ultrasound was also performed (*n* = 47).

The biochemical parameters were measured enzymatically by an automatic analyzer (Cobas Mira Plus; Hoffmann La Roche). Low-density lipoprotein cholesterol (LDL) was calculated according to the Friedewald equation. TSH (*n* = 91), T (*n* = 96), LH (*n* = 74), FSH (*n* = 74), E2 (*n* = 46), and prolactin (*n* = 92) levels were determined in the participants. Testosterone and estradiol levels were measured with commercially available DELFIA kits (DELFIA; Perkin Elmer, Wallac Oy, Turku, Finland). Analytical sensitivity for the testosterone was 0.3 nmol/l, and for estradiol, it was 0.05 nmol/l; the intra-assay variations of the kits were 5.6% and 5.9%, respectively, whereas the interassay variations were 6.8% for testosterone and 5.1% for estradiol. Gonadotropins, prolactin, and TSH were measured with IRMA kits (Beckman-Coulter Inc., France/Czech). For FSH and LH, the intra-assay variations were 4.05% and 7.33%, and interassay variations were 8.2% and 8.42%, respectively, whereas the analytical sensitivity was 0.17 mIU/ml for FSH and 0.16 mIU/ml for LH. For the serum prolactin and TSH, the analytical sensitivity was 15.2 mIU/l and 0.04 mIU/l, intra-assay CV ≤ 2.8% and CV ≤ 3.7%, and interassay CV ≤ 8% and CV ≤ 8.6%, respectively.

In case of consistent symptoms and repeatedly low testosterone levels (<11 nmol/l), additional tests were made to prove the hypogonadism and to found out the possible reasons (free testosterone calculation, gonadotropins to discriminate primary/LH, FSH—above the upper reference range/or secondary hypogonadism/LH, FSH—below the upper reference range/, MRT of the pituitary, karyotype etc.) according to the published guidelines [13–15]. In case of persistent hyperprolactinemia, the diagnostic approach was focused on exclusion of secondary causes such as medication or nutritional supplements, hypothyroidism, liver or kidney dysfunction, other stress factors, and pituitary mass (MRT) [16, 17].

### 2.2. Obesity and Metabolic Syndrome Criteria

Body mass index (BMI) was calculated according to the well-known formula: BMI = weight (kg)/height (m^2^). Metabolic syndrome was diagnosed in the presence of any three of the following five criteria: (1) elevated waist circumference of ≥94 cm; (2) elevated triglycerides of ≥1.7 mmol/l or drug treatment for elevated triglycerides; (3) decreased HDL-ch of <1.03 mmol/l or drug treatment for decreased HDL-ch; (4) elevated fasting glucose of ≥5.6 mmol/l or drug treatment for elevated glucose; and (5) systolic blood pressure of ≥130 mmHg and/or diastolic blood pressure of ≥85 mmHg and/or antihypertensive therapy [18].

### 2.3. Missing Data

In case of missing waist circumference values, a BMI ≥ 30 were used as a marker of obesity (http://www.idf.org). If the available data did not allow a definitive MS positive or negative classification, then the patients (*n* = 7) were excluded from analysis as in other studies [19]. In patients with clear medication-induced GM, testosterone and prolactin levels were rarely measured, and thus, testosterone and prolactin values are available in 96 and 92 of the patients, respectively. Estradiol levels were measured usually in case of suspected gonadal tumor and were available in only 46 patients.

### 2.4. Statistics

Descriptive statistics and frequency analysis were used where appropriate. Comparisons between subjects were performed using chi-square or Fisher's exact test for categorical variables. Correlation analyses were performed with Spearman's rank correlation test. Comparisons between groups were made through Mann–Whitney or Kruskal–Wallis test. *p* values of ≤0.05 were accepted as statistically significant. The data were analyzed by MedCalc Software for Windows (version 15.0; MedCalc Software, Ostend, Belgium).

## 3. Results

A total of 110 males (18–91 years; median, 32.5 years) with GM were included in the study. In 14.5% of the patients, the GM was unilateral, whereas in the other 85.5%, it was bilateral. The duration of the GM varied between one month and 20 years (median, 2 years). A total of 44 of men (40%) were with hypogonadism. Hypergonadotropic hypogonadism was found in 14 patients; 6 of them being with mosaic or complete Klinefelter syndrome. Hypogonadotropic hypogonadism was established in 30 patients; in 12 of them, the gonadotropin and testosterone decrease was caused by prolactinoma, and in 5 of them, the decrease was caused by other pituitary formations. A total of 4 patients were with Kallmann syndrome, other 5 patients were with idiopathic hypogonadotropic hypogonadism, and 1 patient was diagnosed with Bardet–Biedl syndrome. A total of 22 patients (20.0%) were with medication-induced GM due to the use of spironolactone, bicalutamide, dutasteride, chemotherapy, ACE-inhibitors, allopurinol, statins, anabolic steroids, and alcohol, but in some patients on multitherapy, the specific provoking drug could not be identified. In four patients, the GM results from other reasons: leidigoma/*n* = 2/, hepatic injury/*n* = 1/, and thyroid dysfunction/*n* = 1/. In the other 40 patients (36.4%), the cause for GM was not identified. In 18 of these patients, GM appeared during puberty/persistent pubertal GM/, whereas in the other 22 patients, the GM developed in the adulthood/idiopathic postpubertal GM/. Hyperestrogenemia was found in 20 of these patients (50%), whereas mild hyperprolactinemia (prolactin less than twice above the upper referent limit) was found in 10 of them (25%); however, no cause for these alterations had been established. A total of 6 patients (5.5%) admitted use of anabolic steroids, and/or nutritional supplements, but a clear temporal relationship with the development of GM was proven in only two of them. Only four patients admitted alcohol use, whereas 24.5% of the men were current smokers or exsmokers. In 16.4% of patients, more than one cause for GM might be suspected; thus, the most likely cause according to the temporal relationships was accepted as the leading etiological factor. For instance, a patient with a congenital secondary hypogonadism on androgen replacement therapy started to use additionally anabolic steroids to increase his muscle mass and developed symptomatic GM. The prevalence of different GM causes and hormonal parameters of the patients are shown on Table 1.

Only five of the hypogonadal patients (11.4%) were on testosterone treatment, whereas others did not receive hormone replacement therapy because of newly diagnosed disease or noncompliance to the therapy. In 20 of the hypogonadal patients (45.5%), GM was among the primary symptoms leading to the proper diagnosis. A history of cryptorchidism was present in 10 patients (9.1%) with hypogonadism, but in none of the eugonadal patients (*p* < 0.001). All men with history of cryptorchidism were with bilateral GM.

Medication-induced GM was a rare reason for GM in patients younger than 40 years (2.8%) but turned out to be the most frequent cause for the complaints after the same age (55.6%).

The prevalence of family history for diabetes and hypertension among GM patients was 16.4% and 23.6%, respectively; no statistically significant differences between eugonadal and hypogonadal groups were established (*p* > 0.05 for both). In eugonadal idiopathic GM patients and hypogonadal GM patients, the HDL-cholesterol and LDL-cholesterol levels were similar (*p* > 0.05 for both), but the triglyceride concentrations were significantly increased in hypogonadal compared with eugonadal men (1.36 [0.42–4.06] vs. 0.94 [0.42–4.11]; *p*=0.013). The prevalence of hypertension, metabolic syndrome, and diabetes type 2 differed significantly among different groups of patients with GM, whereas the prevalence of obesity was similar among them (Table 2). In patients younger than 40 years, the prevalence of MS was 20% in the idiopathic GM group, 43.8% in men with persistent pubertal GM, 47.6% in patients with a secondary hypogonadism, and 50% in patients with primary hypogonadism. The clinical and hormonal characteristics of patients with GM and MS are shown on Table 3.

In the whole GM group, the levels of testosterone were positively related to the hemoglobin (*r* = 0.423; *p* < 0.001) and hematocrit (*r* = 0.471; *p* < 0.001) levels, as well as negatively associated with the total cholesterol (*r* = −0.225; *p*=0.032), LDL cholesterol (*r* = −0.252; *p*=0.029), and triglyceride concentrations (*r* = −0.226; *p*=0.032). The testosterone levels were not associated with the age, BMI, waist circumference, blood pressure levels, or other metabolic parameters of the patients (*p* > 0.05 for all). Estradiol to testosterone ratio was positively related to leucocytes (*r* = 0.500; *p* < 0.001), waist circumference (*r* = 0.365; *p*=0.028), and triglycerides (*r* = 0.500; *p*=0.001). In males with idiopathic GM, testosterone levels were significantly associated with hemoglobin (*r* = 0.360; *p*=0.031), BMI (*r* = −0.335; *p*=0.046), and HDL levels (*r* = 0.601; *p*=0.001), whereas in other GM patients, the androgen concentration was related to the total (*r* = −0.317; *p*=0.015) and LDL (*r* = −0.353; *p*=0.015) cholesterol as well as to hemoglobin (*r* = 0.357; *p*=0.005).

## 4. Discussion

The present study was focused on the causes of GM in adult individuals and on the metabolic disturbances in that specific group of patients. Results showed that in almost two-third of patients, GM was of pathological origin. Moreover, the development of GM was among the primary symptoms leading to the proper diagnosis in more than 40% of hypogonadal patients. These results supported the conclusions of the largest Danish study on GM which emphasized on the need of structured and detailed investigations of the adults with GM [11].

The relatively higher percentage of pathological findings including hyperprolactinemia and hypogonadism in our group was related to the specific centralized organization of medical services in the country. Most of the patients were referred from general practitioners or endocrinologists, and thus, the patients were admitted to our tertiary endocrine center in case of a suspected endocrine disease or lack of other obvious reasons requiring investigations by other specialists (e.g. drug abuse, renal or hepatic insufficiency, breast cancer, antiandrogen use in prostate cancer patients etc). On the other hand, some young men with idiopathic GM considered the condition as a primarily cosmetic problem and sought consultation with a plastic surgeon only. Therefore, some pathologic GM causes and the percentage of idiopathic GM might be underrepresented in our GM group.

Most of the investigated adult patients presented with bilateral GM, as in a former study on adolescent boys in the same population [6]. In opposite, the prevalence of unilateral and bilateral GM in other ethnic GM groups was almost similar [20].

Interestingly, only 5.5% of our patients recognized anabolic steroid use, whereas no one admitted use of marihuana. The reported prevalence of anabolic steroids use in other adult GM groups was significantly higher (12.9%–13.9%) [11, 20]. Thus, the unexplained hyperestrogenemia in some of our patients might be related to unreported anabolic or drug abuse. Other studies have shown that more than a half of anabolic steroid users would not disclose their substance abuse to any physician, which coincides with our impression [21]. Other possible explanations for the unexplained hyperestrogenemia might be an idiopathic increase of aromatase activity in some patients, obesity and an intake of unknown endocrine disruptors.

The local imbalance between the free estrogens and androgens in the breast is paramount for the development of GM [2]. At the same time, the estradiol to testosterone ratio in the circulation might modulate the risk for the metabolic syndrome development in men [22]. Therefore, it is interesting to find out if the hormonal imbalance in GM individuals might be related to metabolic disturbances. The prevalence of metabolic syndrome among the investigated GM patients was 53%, which was higher than the estimated prevalence of metabolic syndrome in the common male Bulgarian population at similar age (40.9%) [23]. The prevalence of metabolic syndrome was increased in the patients with medication-induced GM and in the hypogonadal patients, whereas it was lowest in the group of men with idiopathic postpubertal GM despite the similar degree of obesity. Even in patients younger than 40 years, the prevalence of MS in hypogonadal GM men was twice as high as that in those with idiopathic GM.

The increased prevalence of MS in patients taking different drugs has been expected because many medications with known side effects on male breast have been used for the prevention or treatment of metabolic and cardiovascular complications. However, the increased prevalence of MS in hypogonadal GM patients and in men with persistent pubertal GM needs further research. The prevalence of metabolic syndrome was strongly increased (44% vs. 10%), whereas insulin sensitivity was decreased in men with Klinefelter syndrome (KS) compared with healthy controls [24]. On the other hand, the prevalence of MS was slightly (but not significantly) increased in KS patients in comparison to 46XY men with nonobstructive azoospermia and men with obstructive azoospermia (34.3%, 23.3%, and 22.2%, respectively) [25]. The presence of metabolic syndrome was also increased in patients with congenital hypogonadotropic hypogonadism compared with healthy men (1.5% vs. 0.25%). However, the prevalence of MS in that group of hypogonadal patients was very low in comparison to the published data for men with primary hypogonadism [26]. In opposite, our data showed increased prevalence of MS in young hypogonadal men with GM, irrespective of the cause of hypogonadism. Further studies are needed to reveal whether the metabolic risk is increased in hypogonadal patients with GM in comparison to those without GM.

Our data showed a strongly increased estradiol to testosterone ratio in GM patients with MS in comparison to those without the syndrome, which correlated to visceral obesity and hypertriglyceridemia in the patients. Moreover, the higher testosterone levels in the patients with GM were associated with a better lipid profile, whereas the androgen influence on other MS components was inconclusive. Some authors reported a negative correlation between testosterone levels and BMI, suggesting increased testosterone to estradiol conversion in the fat tissue of patients with GM [27]. However, other authors did not support such relationships [28]. The positive associations between the testosterone levels and lipid profile were already shown in the common male population [29], in ageing males with andropausal symptoms [30] and in patients with coronary artery disease [31]. On the other hand, increased estradiol levels in men were associated with increased risk of obesity, metabolic syndrome, and diabetes type 2 [32–34]. Moreover, elderly men with prevalent coronary heart disease, heart failure, or stroke had significantly increased estradiol to testosterone ratio in comparison to those without cardiovascular diseases [35]. According to our results, the hormonal imbalance in GM patients might be associated with metabolic disturbances. The increased GM prevalence in hospitalized men with diabetes mellitus supported indirectly this conclusion [36]. However, further longitudinal studies are needed to evaluate the long-term consequences of sex hormones changes on the cardiovascular morbidity and mortality of adults with GM.

To the best of our knowledge, this is the first study focused on metabolic disturbances in adult men with GM due to different causes. However, some limitations should be mentioned. GM established by palpation was not confirmed by ultrasonography or X-ray mammography in all men. Additionally, patients with incomplete laboratory and hormonal data were included in the study to avoid preselection bias. The analysis of the missing data showed that standardized approach with detailed laboratory investigations and imaging studies was commonly applied to patients with newly developed GM and in case of concomitant symptoms suggesting pathologic condition (e.g. hypogonadism, hyperprolactinemia, systemic disease etc). On the contrary, patients with long-lasting pubertal GM without additional symptoms and those with a plausible explanation for GM (e.g. use of spironolactone) were rarely an object of further investigations. However, the recently published guideline for the evaluation and treatment of GM emphasizes on the need of thorough investigations, even in case of an apparent reason for GM in adults [37]. The implementation of that principle in the clinical practice might help early diagnosis of some endocrine and oncological diseases.

In conclusion, our study showed that the development of GM in adults might be an important symptom of an underlying gonadal disease. Moreover, it could be associated with an increased risk of metabolic disturbances. Our results support the need of detailed laboratory and hormonal investigations in patients with GM. Moreover, the targeted screening for lipid and carbohydrate disturbances in males with GM might reduce the potential cardiovascular risk related to the hormonal imbalance in these patients.

## Figures and Tables

**Table 1:** Causes for gynecomastia in different patients and hormonal characteristics in the investigated group. Data are presented as median (*n*), min–max. ^*∗*^low prolactin levels in patients already treated with dopamine agonists or transsphenoidal surgery. The minimal prolactin level at the time of prolactinoma diagnosis was 1088 mIU/l. One of the patients was with somatoprolactinoma. ^*∗∗*^indicates normal testosterone levels in patients with already started testosterone therapy. All hypogonadal patients had initial testosterone levels under 11 nmol/l and additional tests to prove hypogonadism. In one patient with panhypopituitarism, the testosterone level was very low but measured in other laboratory/not included/.

Causes for GM	*N*	%	Categories	Age (years)	Testosterone (nmol/l)	LH (IU/l)	FSH (IU/l)	Prolactin (mIU/l)	TSH (mIU/l)	Estradiol (pmol/l)
Persistent pubertal GM	18	16.4	Persistent pubertal GM	22.00 (18) 18–40	20.90 (15) 9.70–30.60	2.85 (12) 0.60–9.70	2.30 (13) 1.40–7.60	308.00 (14) 91.00–652.00	1.80 (15) 0.87–26.80	394.00 (11) 112.00–633.00

Idiopathic postpubertal GM	22	20.0	Idiopathic postpubertal GM	28.00 (22) 19–66	18.60 (21) 11.40–40.20	2.55 (16) 0.47–7.70	4.04 (15) 0.53–8.30	214.00 (21) 82.00–430.00	2.45 (20) 0.62–6.90	282.00 (14) 23.00–960.00

Secondary hypogonadism	18	16.4	Secondary hypogonadism	28.00 (18) 18–69	2.60 (17) 0.60–17.90^*∗∗*^	1.02 (14) 0.47–3.50	1.45 (14) 0.60–3.40	160.00 (15) 108.00–559.00	2.30 (15) 1.00–15.80	304.00 (6) 178.00–761.00

Prolactinoma	12	10.9	Prolactinoma	31.50 (12) 23–53	12.50 (11) 3.20–35.10	0.80 (5) 0.60–2.10	2.40 (5) 1.20–6.00	474.00 (12) 82.00^*∗*^–10561.00	2.00 (8) 0.51–4.20	248.00 (1)

Primary hypogonadism	14	12.7	Primary hypogonadism	33.00 (14) 21–67	5.05 (14) 0.60–10.80	12.65 (14) 6.70–29.00	25.71 (14) 13.50–56.50	191.00 (14) 72.00–667.00	1.80 (13) 0.95–4.40	467.50 (6) 110.20–631.00

Medication-induced GM	20	18.2	Medication-induced GM	60.00 (20) 33–91	14.30 (13) 4.50–44.20	3.30 (9) 2.00–29.90	6.70 (9) 2.00–47.00	286.00 (11) 137.00–602.00	2.10 (16) 0.90–4.80	332.50 (6) 129.00–1171.00

Testicular tumor	2	1.8	Other causes	34.00 (6) 18–44	8.50 (5) 1.40–22.90	2.10 (4) 0.80–3.50	4.35 (4) 1.30–7.40	309.00 (5) 110.00–1030.00	0.39 (4) 0.03–1.40	997.00 (2) 747.00–1247.00
Hepatic injury	1	0.9								
Thyreotoxicosis	1	0.9								
Anabolic steroid abuse	2	1.8								

All	110	100.0	Referent ranges		8.5–42	2–8	3–12	<350	0.3–4.0	<180

**Table 2:** Prevalence of obesity, hypertension, prediabetes (impaired fasting glucose and/or impaired glucose tolerance), metabolic syndrome (MS), and diabetes mellitus type 2 (DM2) in patients with GM due to different causes. The prevalence of obesity, hypertension, prediabetes, MS, and DM2 was compared among the different GM groups through Pearson *χ*^2^ test, *p* < 0.05 considered statistically significant. Data are presented as % (*n*). The presence of MS in 6 patients and the presence of obesity in 3 patients were not established due to missing metabolic or anthropometric data.

GM cause	Obesity % (*N* = 103)	Hypertension % (*N* = 106)	Prediabetes % (*N* = 106)	DM2 % (*N* = 106)	MS % (*N* = 100)
GM group (*N* = 106)	**37.9 (39)**	**34.9 [37]**	**13.2 [14]**	**15.1 [16]**	**53 (53)**
Persistent pubertal GM (*N* = 18)	38.9 (7)	38.9 (7)	11.1 (2)	0 (0)	47.1 (8)
Idiopathic postpubertal GM (*N* = 22)	31.8 (7)	22.7 (5)	18.2 (4)	4.5 (1)	35.0 (7)
Secondary hypogonadism (*N* = 30)	37.9 (11)	23.3 (7)	10.0 (3)	10.0 (3)	46.7 (14)
Primary hypogonadism (*N* = 14)	35.7 (5)	21.4 (3)	21.4 (3)	7.1 (1)	57.1 (8)
Medication and AAS-induced GM (*N* = 22)	45 (9)	68.2 (15)	9.1 (2)	50 (11)	84.2 (16)
*p*	0.937	**0.004**	0.742	**<0.001**	**0.028**

**Table 3:** Clinical and hormonal characteristics of GM patients with and without metabolic syndrome (patients with medication-induced gynecomastia were excluded from analyses). Data are presented as median [min–max] or percentage (*n*). Differences between groups were established through Mann–Whitney test or Fisher's exact test, *p* < 0.05 considered statistically significant (^*∗*^).

	Metabolic healthy (*N* = 47)	Metabolic syndrome (*N* = 38)	*p*
Age (years) *n* = 85	27 [18–69] (*n* = 47)	32 [18–67] (*n* = 38)	0.078
Family history for DM2 (%) (*n* = 85)	8.5% (4) (*n* = 47)	21.1% (8) (*n* = 38)	0.124
Family history for AH (%) (*n* = 85)	21.3% (10) (*n* = 47)	28.9% (11) (*n* = 38)	0.456
Obesity (%) (*n* = 84)	25.5% (12) (*n* = 47)	45.9% (17) (*n* = 37)	0.066
Testosterone (nmol/l) (*n* = 78)	13.00 [0.60–35.10] (*n* = 44)	9.25 [0.60–30.60] (*n* = 34)	0.148
Estradiol (pmol/l) (*n* = 37)	275.00 [23.00–585.00] (*n* = 19)	357.50 [107.00–960.00] (*n* = 18)	0.081
Prolactin (mIU/l) (*n* = 76)	227.50 [82.00–5240.00] (*n* = 44)	191.50 [72.00–10561.00] (*n* = 32)	0.245
TSH (mIU/l) (*n* = 72)	1.85 [0.03–6.9] (*n* = 38)	2.20 [0.62–26.80] (*n* = 34)	0.119
LH (IU/l) (*n* = 61)	2.30 [0.47–22.40] (*n* = 35)	2.55 [0.47–29.00] (*n* = 26)	0.924
FSH (IU/l) (*n* = 60)	3.0 [0.53–38.90] (*n* = 35)	2.8 [0.77–56.50] (*n* = 25)	0.514
Estradiol (pmol/l) to testosterone (nmol/l) ratio ^*∗*^(*n* = 36)	13.14 [1.65–90.00] (*n* = 18)	56.03 [7.23–691.82] (*n* = 18)	0.001

## Data Availability

Datasets are available from the corresponding author on reasonable request after permission from the local authorities.
